# Persistent organic pollutants in muscle of fish collected from the Nové Mlýny reservoir in Southern Moravia, Czech Republic

**DOI:** 10.1007/s10661-015-4460-3

**Published:** 2015-06-20

**Authors:** Lenka Zelníčková, Zdeňka Svobodová, Petr Maršálek, Radka Dobšíková

**Affiliations:** Department of Veterinary Public Health, Animal Protection and Welfare, University of Veterinary and Pharmaceutical Sciences Brno, Palackeho tr. 1/3, 612 42 Brno, Czech Republic

**Keywords:** POPs, Organochlorine pesticides, Polychlorinated biphenyls, Carp, Bream, Pike perch

## Abstract

The aim of the present study was to investigate the content of organochlorine pesticides (OCPs) and polychlorinated biphenyls (PCBs) in muscle tissues of fish, i.e. carp (*Cyprinus carpio*, L.), bream (*Abramis brama*, L.) and pike perch (*Stizostedion lucioperca*, L.) from the middle Nové Mlýny reservoir and compare our results with previous corresponding studies. Samples were analysed by gas chromatography with ion trap tandem mass spectrometry. The highest contents of all pollutants were determined in muscle tissue of bream. The analysis of HCHs showed that *β*-HCH was the most abundant. PCB congener 28 was evaluated as the predominant PCB congener. The metabolite *p*,*p*’-DDE exhibited the highest concentration of all the monitored metabolites. The monitoring confirmed significantly (*P* < 0.01) higher concentrations of OCP and PCB in muscle of bream compared to carp and pike perch. The contents of pollutants in our study were found to be lower in comparison to the findings of some previous studies.

The aquatic ecosystem is contaminated with many organic and inorganic substances, including agricultural and industrial pollutants. For the evaluation of environmental contamination, different bioindicators of animal or plant origin are used. Fish are one of the most important bioindicators used to assess the rate and degree of aquatic pollution.

Persistent organic pollutants (POPs), such as polychlorinated biphenyls (PCBs), dichlorodipenthyltrichloroethane (DDT), hexachlorobenzene (HCB) and hexachlorocyclohexane (HCH), are a global problem in the area of environmental pollution due to their toxicity and long-term persistence in the environment. All POPs have a negative effect on the flora and fauna in affected areas (Naccari et al. 2004). These toxic substances accumulate in the food chain, and many of them are potentially carcinogenic and mutagenic. DDT and its metabolites are also known as endocrine disruptor chemicals (EDCs) and may cause dysfunctions of the nervous and reproductive systems of aquatic organisms, wildlife and people (Thomas et al. 2012).

PCBs are a group of chlorinated hydrocarbons and were originally used, due to their high chemical and physical stability, in the manufacturing of electrical equipment, e.g. as a filling in condensers, heat-exchangers, hydraulic fluids and lubricants, and in many other applications up to the late 1970s (Marsalek et al. 2004). The main sources of PCBs in the ecosystem are soils, in which more than 90 % of these substances are deposited (Lana et al. 2008). Because of the negative effects of PCBs and due to mass poisonings in Japan and Taiwan, their production was reduced and at the end of 1970s, they were banned (Romanic et al. 2012) in the USA and many developed countries, including the Czech Republic. Nevertheless, PCBs persist in all parts of the environment, and their half-lives vary from months to decades (Gallo et al. 2011). PCBs consist of 209 congeners, which can be separated into groups according their persistence, structure and toxicological properties (Gallo et al. 2011).

The aim of this study was to determine the content of 11 organochlorine pesticides (OCPs) and 7 PCB congeners in fish tissues (carp, bream and pike perch) from the middle Nové Mlýny reservoir and compare the results with some previous corresponding studies (Hajslova et al. 1997; Marsalek et al. 2004).

## Materials and methods

**Fig. 1:**
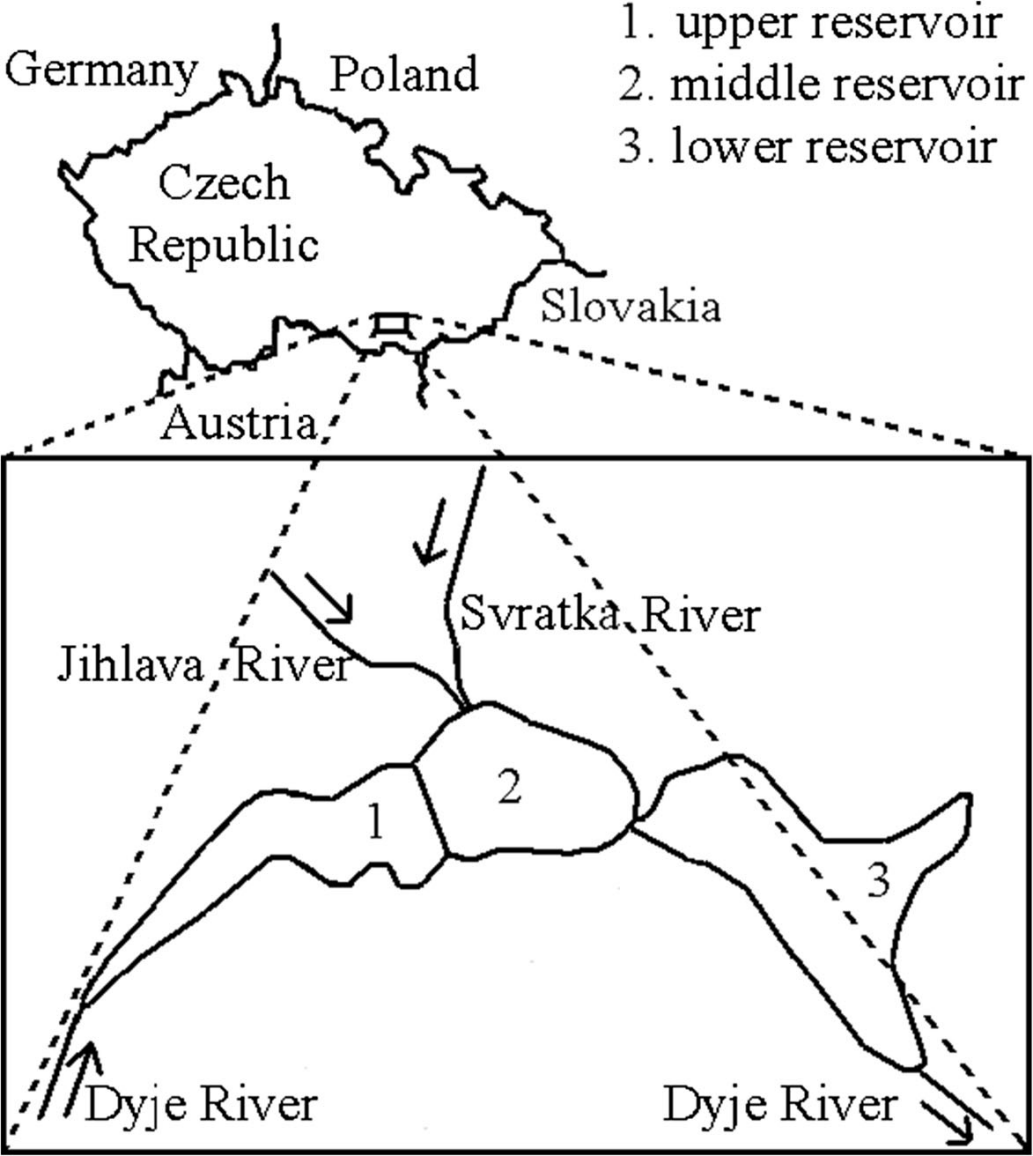
Map of the Czech Republic and location of sampling site

The Nové Mlýny reservoirs are located in South Moravia and form a cascade of three reservoirs with an area of 32.3 km^2^ (the upper Mušovská, the middle Věstonická and the lower Novomlýnská) on the Dyje River. The Svratka and Jihlava Rivers flow into the middle reservoir (Fig. 1). Due to their location, they are often monitored. All three rivers run through important agriculture areas of Southern Moravia. In contrast to Dyje River, which flows into the upper reservoir, the Svratka and Jihlava Rivers flow through big industrial cities of Southern Moravia (e.g. Brno, Blansko, Trebic, Jihlava). Thus, Věstonická reservoir was chosen as the Svratka and Jihlava Rivers, potential pollution sources, flow into it.

Altogether, 29 fish samples collected using fishnets from the middle reservoir, i.e. Věstonická, were examined. Samples of the cranial parts of dorsal muscles (approx. 70 g) of 10 carp, 11 bream, and 8 pike perch were taken in September and October 2013. The biometric characteristics of fish are given in Table 1. The samples were analysed for the content of OCPs, i.e. the content of DDT and its metabolites, defined as the sum of *p*,*p*’-DDT, *p*,*p*’-DDE, *p*,*p*’-DDD, *o*,*p*’-DDT, *o*,*p*’-DDE and *o*,*p*’-DDD, and the content of HCHs, defined as the sum of *α*-, *β*-, *γ*- and *δ*-HCH, and HCB in μg kg^−1^ of fish muscle of wet weight. In the study, also PCB congeners, defined as the sum of indicator congeners according to IUPAC No. 28, 52, 101, 118, 138, 153 and 180, were monitored.

**Table 1:** Biometric characteristics of fish

Fish	*N*	Age (years) Mean (min–max)	Fat content (%) Mean ± SD	Body weight (g) Mean ± SD
Carp	10	6.6 (5–8)	1.0 ± 0.9	2552.1 ± 316.2
Bream	11	6.2 (3–8)	6.6 ± 4.6	1866.3 ± 485.4
Pike perch	8	6.4 (4–10)	1.0 ± 0.3	3392.9 ± 1547.6

The qualitative and quantitative analyses of OCPs and PCBs were performed by gas chromatography with ion trap tandem mass spectrometry (GC/MS). Marsalek et al. (2013) described the analytical procedure used for the analysis of OCPs and PCBs in muscle tissue. Approximately, 50 g of a representative portion of muscle tissue was homogenized and extracted into diethylether (2 × 100 ml). The extract was dried using anhydrous sodium sulphate and evaporated with a rotary vacuum evaporator. After evaporation of the solvent, the lipid residues were determined gravimetrically. An aliquot of isolated lipid (0.2 ± 0.05 g) was dissolved in *n*-hexane (2 × 4 ml) and cleaned on a Florisil packed column (90 ml of a *n*-hexane:diethylether mixture, 94:6, *v*/*v*). The cleaned solution was concentrated by a rotary vacuum evaporator and, after evaporation of the solvent, the fraction was redissolved in cyclohexane, of which 2 ml were used for analysis. The separation, identification and quantification of OCPs and PCBs were carried out using a Varian 450-GC gas chromatograph with a Varian 220-MS ion trap mass spectrometer and VF-5 ms (30 m × 0.25 mm) column (Varian, Inc., USA).

Statistical analysis was performed using Unistat 5.6. software. The results were tested for normality using the Shapiro-Wilk test. Data on ΣHCH, ΣPCB and ΣDDT were log-transformed, and data on HCB were square-root-transformed to improve the normal distribution. Individual differences among the means were tested using the Tukey-HSD test. Non-parametric Spearman correlation was used for comparing the relationships among the analysed parameters. In the cases where the content of an organic pollutant was not detected, half of the detection limit was applied for statistical analysis (Table 2). Significance for all parameters was set at *P* < 0.05 or *P* < 0.01.

**Table 2:** List of all measured organochlorines with their detection limits (μg kg^−1^)

Substance	DL	Substance	DL	Substance	DL
PCB 28	0.06	*p*,*p*’-DDT	0.20	*α*-HCH	0.10
PCB 52	0.03	*o*,*p*’-DDT	0.13	*β*-HCH	0.08
PCB 101	0.05	*p*,*p*’-DDE	0.20	*γ*-HCH	0.08
PCB 118	0.20	*o*,*p*’-DDE	0.15	*δ*-HCH	0.06
PCB 138	0.06	*p*,*p*’-DDD	0.13	HCB	0.06
PCB 153	0.07	*o*,*p*’-DDD	0.38		
PCB 180	0.04				

*DL* detection limit

## Results

The results of organic pollutant contents in muscle tissues, including arithmetic mean, standard deviation and Spearman rank correlation, are given in Table 3. Statistical differences of the POPs contents across fish species within a contaminant are provided.

**Table 3:** Contents of POPs (μg kg^−1^ w.w.) in muscle tissue

Fish		ΣHCH	HCB	ΣPCB	ΣDDT
Carp	Mean ± SD	0.1 ± 0.06^b^	0.2 ± 0.1^b^	6.6 ± 3.7^b^	32.1 ± 18.7^b^
	Median	0.09	0.1	6.2	28.4
	*r*_s_ fat content	**0.78**	**0.56**	**0.99**	**0.66**
	*r*_s_ age	0.32	−0.01	0.53	0.09
	*r*_s_ body weight	0.22	0.42	0.08	0.38
Bream	Mean ± SD	0.49 ± 0.45^a^	2.6 ± 1.8^a^	70.3 ± 60.1^a^	373.9 ± 258.6^a^
	Median	0.3	2.4	68.1	343.0
	*r*_s_ fat content	**0.92**	**0.96**	**0.99**	**0.89**
	*r*_s_ age	0.17	0.16	0.11	0.35
	*r*_s_ body weight	**0.73**	**0.53**	**0.65**	**0.80**
Pike perch	Mean ± SD	0.09 ± 0.08^b^	0.1 ± 0.08^b^	8.8 ± 7.0^b^	34.5 ± 33.2^b^
	Median	0.07	0.07	6.6	22.7
	*r*_s_ fat content	**0.86**	0.57	0.41	**0.74**
	*r*_s_ age	0.11	0.52	0.35	−0.19
	*r*_s_ body weight	0.07	0.48	0.33	0.24

*r*_s_ Spearman rank correlation (Correlation coefficients are highlighted as a bold text in cases when Spearman rank correlation is significant (*P* < 0.05), *SD* standard deviation

### HCH isomers

HCH isomers were detected in 63.8 % of all samples (75 % in the case of bream >72.5 % in the case of carp >43.8 % in the case of pike perch). The content of ΣHCH in muscle of carp ranged from 0.04 to 0.2 μg kg^−1^ w.w.; in bream, from 0.08 to 1.5 μg kg^−1^ w.w.; and in pike perch, from 0.05 to 0.3 μg kg^−1^ w.w. The content of HCH and its isomers decreased in the following order: *β*-HCH > *α*-HCH > *δ*-HCH ∼ *γ*-HCH. Significant correlations (*P* < 0.01) were found between HCH content and fat content in muscle of carp (0.78), bream (0.92) and pike perch (0.86), and a significant correlation (*P* < 0.01) between HCH content and the body weight of bream (0.73).

### HCB

HCB was detected in 96.6 % of samples. The highest HCB content was found in bream and the lowest in pike perch. The content of HCB in muscle of carp ranged from 0.03 ng kg^−1^ to 0.3 μg kg^−1^ w.w.; in bream, from 0.3 to 6.0 μg kg^−1^ w.w.; and in pike perch, from 0.04 to 0.3 μg kg^−1^ w.w. A significant correlation (*P* < 0.05) between HCB content and fat content in carp (0.56) and a significant correlation (*P* < 0.01) between HCB content and fat content in bream (0.96) were found. Moreover, a significant correlation (*P* < 0.05) between HCB content and body weight in muscle of bream (0.53) was also detected.

### PCB congeners

PCB congeners were observed in the majority of analysed samples (96.6 %). In the cases of bream and pike perch, PCB congeners were found in all 19 samples. The content of ΣPCB in muscle of carp ranged from 2.4 to 14.1 μg kg^−1^ w.w.; in bream, from 8.0 to 210.3 μg kg^−1^ w.w.; and in pike perch, from 3.1 to 24.8 μg kg^−1^ w.w. The PCB congeners decreased in the following order: PCB 28 > PCB 138 > PCB 180 > PCB 101 > PCB 153 > PCB 118 > PCB 52. We also observed significant correlations (*P* < 0.01) between PCB content and fat content in muscle of carp and bream (both 0.99), and significant correlations (*P* < 0.05) between PCB content and the body weight of bream (0.65).

### DDT and its metabolites

DDTs were found in 93.7 % of the analysed samples. The metabolites *p*,*p*’-DDE, *p*,*p*’-DDD, *o*,*p*’-DDT and *o*,*p*’-DDD were determined in all samples of carp, bream and pike perch. The content of ΣDDT in muscle of carp ranged from 10.8 to 61.1 μg kg^−1^ w.w.; in bream, from 53.9 to 790.0 μg kg^−1^ w.w.; and in pike perch, from 16.0 to 115.2 μg kg^−1^ w.w. The content of DDT and its metabolites decreased in the following order: *p*,*p*’-DDE > *o*,*p*’-DDT > *p*,*p*’-DDD > *o*,*p*’-DDD > *p*,*p*’-DDT ∼ *o*,*p*’-DDE. In the analysis of DDT, its metabolite *p*,*p*’-DDE exhibited the highest content (the mean value was 233.0 μg kg^−1^ w.w.) of all the monitored metabolites. Significant correlations (*P* < 0.05) were found between DDT content and fat content in muscle of carp (0.66) and pike perch (0.71). A significant correlation (*P* < 0.01) between DDT content and fat content in muscle of bream (0.89) and a significant correlation (*P* < 0.01) between DDT content and the body weight of bream (0.80) were also found.

## Discussion

Chemical monitoring is one of the best potential possibilities for assessing environmental contamination. Fish are often used for the assessment of aquatic pollution.

Our monitoring confirmed a significant correlation between OCP content and fat content in the muscle of all fish tested, and between OCP content and the body weight of bream. The monitoring confirmed significantly (*P* < 0.01) higher concentrations of OCP and PCB in muscle of bream compared to carp and pike perch. The obtained results show that the highest contents of all pollutants were detected in muscle of bream. In all samples, HCHs had the lowest contents. This may be due to their lower degree of persistence and lower lipophilicity (Davodi et al. 2011). The analysis of HCH isomers showed that *β*-isomer was the most abundant and was found in all analysed samples (the mean value was 0.4 μg kg^−1^ w.w.). PCB congener 28 was evaluated as the most abundant of all the PCB congeners (the mean value was 24.4 μg kg^−1^ w.w.).

The results of our monitoring from the middle Nové Mlýny reservoir, performed in 2013, were compared with the results obtained from the same reservoir in recent years by Hajslova et al. (1997), Vavrova et al. (2003) and Marsalek et al. (2004). Marsalek et al. (2004) compared the time courses of DDT contamination between 1995 and 2002 for individual species. The DDT content showed a significant decrease over the monitoring time. The greatest decrease was found for pike perch (91 %), followed by bream (75 %) and by carp (35 %). The authors also found that the highest PCB content was in muscle of bream (305 μg kg^−1^ w.w.), followed by carp (210 μg kg^−1^ w.w.) and pike perch (110 μg kg^−1^ w.w.). Hajslova et al. (1997) described the same trend. The authors conducted long-term monitoring of PCB contents in fish from Czech rivers and found that the congeners PCB 153 and PCB 180 had the highest contents. The highest PCB and DDT contents were found in the case of common bream fillets (75 and 167 μg kg^−1^w.w., respectively). Vavrova et al. (2003) detected PCB contents in muscle of carp, bream, pike, etc. The congeners PCB 153 and PCB 138 had the highest contents in all fish species (in the case of PCB 153: bream, 22.4 μg kg^−1^ w.w. > pike, 13.4 μg kg^−1^ w.w. > carp, 10.4 μg kg^−1^ w.w.; in the case of PCB 138: bream, 13.5 μg kg^−1^ w.w. > pike, 10.0 μg kg^−1^ w.w. > carp 6.8, μg kg^−1^ w.w.). These facts are in agreement with our findings. Contamination in the case of PCB and DDT contents also decreased in the following order: bream > pike perch > carp. Because of fast degradation of low-chlorinated congeners in the environment, an increased level of low-chlorinated PCB 28 congener can be caused by a recent contamination of unknown origin.

In the Commission Regulation (EU) No 1259/2011 amending Regulation (EC) No 1881/2006 as regards maximum levels for dioxins, dioxin-like PCBs and non dioxin-like PCBs in foodstuffs, there is given only a limit for six PCB congeners (sum of PCB 28, 52, 101, 138, 153 and 180, − ICES – 6) in muscle of fish and fishery products and products thereof as 75 μg kg^−1^ w.w. In our study, the sum of 7 PCB congeners analysed (sum of PCB 28, 52, 101, 118, 138, 153 and 180) was found under the Commission Regulation (EU) 1259/2011 limit.

For the limits of DDT and its metabolites, a Czech national legislation was used. In the Notice No 158/2004, on maximum residue limits of pesticides in foodstuff, the limit for DDT in fish is 0.5 mg kg^−1^ w.w. In our study, no result exceeded the limit for DDT. As for PCB congeners and DDT and its metabolites, the highest concentration was found in the muscle tissue of bream. It can be related to higher fat content in bream muscle (6.6 %) when compared to carp (1.0 %) and pike perch (1.0 %) samples. Our results are in accordance to the ones of Marsalek et al. (2004), Hajslova et al. (1997) and Vavrova et al. (2003).

The above findings are also in agreement with other studies from the Czech Republic. Havelkova et al. (2008) found that muscle of brown trout in Králíky on the Tichá Orlice River exhibited the highest content of PCB congeners. In the other study, Havelkova et al. (2007) monitored eight rivers (Orlice, Chrudimka, Cidlina, Jizera, Vltava, Ohre, Bilina and the Blanice River). The chub (*Leuciscus cephalus* L.) was selected as the indicator species. The large majority (70–80 %) of PCB congeners was from the group of higher-chlorinated PCB 138, 153 and 180. The river with the highest DDT pollution was the Ohre (44.01 μg kg^−1^ w.w. muscle). The dominant metabolite of DDT was made up of the metabolite *p*,*p*’-DDE (75–90 %). The levels of other metabolites decreased in the following order DDE > DDD > DDT. The highest HCH concentrations in muscle of chub were found in the Cidlina River. The *β*-HCH and *γ*-HCH (35–50 %) were the most abundant. The least abundant, the *α*-HCH, was found (approx. 15 %). *γ*-HCH has been used in agriculture for its insecticidal effects and is still found in the environment because of its bioaccumulation in soil. In the Elbe River, Randak et al. (2009) detected PCB congeners, DDT and HCB in chub. A study by Hradkova et al. (2012) also documented the contamination of the Elbe River by these substances. Svobodova et al. (2003; 2004) studied the extent of contamination by persistent organic pollutants in tissues of carp from 6 ponds in South and West Bohemia. In these studies, PCB congeners 138, 153 and 180, *p*,*p*’-DDE and *β*-HCH exhibited the highest concentrations of all monitored metabolites at all locations. Lana et al. (2010) found out the extent of pollution of the Svratka River with organochlorinated pollutants. The chub (*L. cephalus*) was used as a suitable bioindicator of the water contamination. The mean values of DDTs, HCHs, HCB and the sum of seven indicator PCB congeners were 29.9, 1.0, 2.2 and 31.7 ng g^−1^ w.w. Their results show a predominance of DDE and higher-chlorinated PCB congeners.

With respect to results from other countries, Macgregor et al. (2010) investigated the concentration of POPs in eels (*Anguilla anguilla*) collected over a 5-year period (2004–2008) from 30 sites across Scotland, including PCBs, DDT and its metabolites, HCH isomers and HCBs. They found *p*,*p*’-DDE and *p*,*p*’-DDD in almost all samples. *β*-HCH and HCB were detected in a high proportion of eel samples (51 and 45 % of all samples, respectively), while *α*-HCH was detected in 2 % of eels. The content of *α*-HCH was very low (<3 μg kg^−1^ w.w. or below detection limits). PCB congeners 153, 138, 118 and 180 were the most prevalent. The authors compared their study with some previous studies and found no temporal changes in the content of PCB congeners in samples of eel from Scottish rivers. They observed a substantial decrease in *p*,*p*’-DDE and a dramatic reduction in *γ*-HCH in comparison with results gained in 1986. A comparison with data recorded in 1995 revealed substantial reductions in *γ*-HCH contents, while *p*,*p*’-DDE and PCB congeners did not show any significant temporal differences. This is in agreement with the findings of our study. Thomas et al. (2012) investigated organochlorine pesticides and polychlorinated biphenyls in sediments and muscles of farmed fish species (*Cyprinus carpio* and *Perca fluviatilis*) from ponds located in north-eastern France (Lorraine Region). *p*,*p*’-DDE exhibited the highest average contents among organochlorines (0.6 ± 0.4 μg kg^−1^ w.w. for carp and 0.6 ± 0.3 μg kg^−1^ w.w. for perch). The content of ΣPCB ranged from 0.3 to 6.4 μg kg^−1^ w.w. in muscle of carp and from 0.9 to 5.6 μg kg^−1^ w.w. in muscle of perch. PCB congener 52 was found to be predominant. HCHs and HCB were detected only in muscle of carp. In the case of ΣHCH, *β* isomer was the most abundant, with a mean value of 0.6 ± 0.2 μg kg^−1^ w.w. A different trend was revealed in the study by Davodi et al. (2011). They also determined organochlorine pesticides and polychlorinated biphenyls in eight fish species (*Barbus sharpeyi*, *Barbus gerypus*, *Barbus barbulus*, *Barbus luteus*, *C. carpio*, *Aspius vorax*, *Ctenopharyngodon idella* and *Liza abu*) from the largest Iranian wetland, the Shadegan Marshes. The highest content of ΣHCH was found in carp (410 ± 180 μg kg^−1^ lipid weight) and *α*-HCH was the predominant isomer. The most frequent PCB congeners in all samples were PCB 28 and 52. The major sources of contamination in this area included the use of pesticides in agriculture and the presence of chemical and petrochemical refineries.

## Conclusions

Although POPs were banned or limited in use approximately 30 years ago, they are still present in the environment. The levels of contamination obtained by chemical monitoring in three different fish species taken from the middle Nové Mlýny reservoir (Vestonicky reservoir) seem to have decreased when compared to previous studies performed in the same reservoir. However, the presence of predominated PCB congener 28 indicates a possible recent contamination. As the occurrence of persistent organic pollutants in the environment represents a global problem, risk elimination strategies should be coordinated on a global level.
